# Zinc Acetate Inhibits Hepatitis A Virus Replication: Possible Treatment for Patients with Type A Acute-on-Chronic Liver Failure

**DOI:** 10.3390/pathogens14090882

**Published:** 2025-09-03

**Authors:** Tatsuo Kanda, Reina Sasaki-Tanaka, Hiroyuki Abe, Takeshi Yokoo, Akira Sakamaki, Kazunao Hayashi, Hiroteru Kamimura, Atsunori Tsuchiya, Ryota Masuzaki, Hirofumi Kogure, Hiroaki Okamoto, Shuji Terai

**Affiliations:** 1 Division of Gastroenterology and Hepatology, Uonuma Institute of Community Medicine, Niigata University Medical and Dental Hospital, Uonuma Kikan Hospital, 4132 Urasa, Minami-Uonuma 949-7302, Japan; 2 Division of Gastroenterology and Hepatology, Graduate School of Medical and Dental Sciences, Niigata University, 1-757 Aasahimachi-Dori, Chuo-ku, Niigata 951-8520, Japansaka-a@med.niigata-u.ac.jp (A.S.);; 3 Department of Gastroenterology and Hepatology, Faculty of Medicine, University of Yamanashi, Chuo 409-3898, Japan; a.tsuchiya@yamanashi.ac.jp; 4 Division of Gastroenterology and Hepatology, Department of Medicine, Nihon University School of Medicine, 30-1 Oyaguchi Kamicho, Itabashi-ku, Tokyo 173-8610, Japan; masuzaki.ryota@nihon-u.ac.jp (R.M.); kogure.hirofumi@nihon-u.ac.jp (H.K.); 5 Division of Virology, Department of Infection and Immunity, Jichi Medical University School of Medicine, 3311-1 Yakushiji, Shimotsuke 329-0498, Japan; hokamoto@jichi.ac.jp

**Keywords:** antiviral, glucose-regulated protein 78, hepatitis A virus, ribavirin, zinc acetate

## Abstract

Hepatitis A virus (HAV) infection sometimes results in the occurrence of acute liver failure and acute-on-chronic liver failure (ACLF), which is often fatal, especially in patients with diabetes mellitus or elderly individuals. ACLF is observed in patients with cirrhosis who occasionally have zinc deficiency. However, effective drugs for hepatitis A are currently unavailable. Glucose-regulated protein 78 (GRP78) is an antiviral agent that has been reported to prevent HAV replication. The effects of zinc acetate on HAV HA11-1299 genotype IIIA replication and changes in GRP78 levels in human hepatocytes with or without HAV infection were examined. Zinc acetate inhibited HAV HA11-1299 genotype IIIA replication in both Huh7 and GL37 cells. Zinc acetate also inhibited HAV replication in both low- and high-glucose media. Zinc acetate increased the expression of GRP78, in response to HAV replication. The combination of zinc acetate with ribavirin led to greater suppression of both HAV HA11-1299 genotype IIIA and HAV HM175/18f genotype IB replication in Huh7 cells than that of ribavirin alone. In conclusion, zinc acetate inhibits HAV replication in accompany with the elevation of GRP78 expression without causing cellular toxicity. Zinc compounds may be useful for the treatment of ACLF caused by HAV infection.

## 1. Introduction

Hepatitis A virus (HAV) infection is a major cause of acute hepatitis with mild to severe symptoms and, rarely, acute liver failure (ALF) and acute-on-chronic liver failure (ACLF), which are often fatal [1]. ACLF is observed in cirrhotic patients who occasionally have zinc deficiency [2]. In 2016, 7134 people died from hepatitis A worldwide [1]. Effective vaccines for hepatitis A have been developed to prevent HAV infection; however, effective drugs for hepatitis A are currently not available. Considering this background information, we decided to investigate the effects of zinc compounds as antiviral agents on HAV replication.

HAV is a positive-sense RNA virus, belonging to the *Picornaviridae* family, and is classified into the *Hepatovius* genus [3]. HAV RNA genome is approximately 7.6 kb in length and encodes structural (VP4, VP2, VP3, and VP1) and nonstructural (2A, 2B, 2C, 3A, 3B, 3C, and 3D) proteins. HAV RNA has an internal ribosomal entry site (IRES), which is composed mainly of a 5′-nontranslational region (5′-NTR) and a 3′-NTR, and HAV proteins are generated via HAV IRES-mediated translation [3].

Zinc compounds, such as zinc oxide nanoparticles, zinc sulfate, and zinc chloride, protect against virus infection [4,5,6,7]. Zinc sulfate and zinc chloride suppress HAV RNA replication in human hepatocytes [4,5,6,7]. We previously demonstrated that Japanese rice-koji extracts, which induce the expression of glucose-regulated protein 78 (GRP78), have an inhibitory effect on HAV replication [8]. GRP78, a cell-protective ER chaperone protein, is involved in the endoplasmic reticulum (ER) stress pathway and is also known as heat shock protein family A member 5 (HSPA5) or binding immunoglobulin protein (BiP). Transcriptome sequencing (RNA-Seq) analysis revealed that miso extracts also affect the zinc homeostasis pathways [8]. Zinc sulfate could inhibit HAV replication in association with increased GRP78 expression [8].

HAV replication is sensitive to the interferon and interferon signaling pathways [9,10]. It has been recently reported that GRP78 can enhance the activation of the interferon/Janus kinase (JAK)-signal transducer and activator of transcription (STAT) pathway during influenza A virus infection [11]. In the present study, the effects of zinc acetate on HAV replication were examined and the changes in GRP78 levels were also investigated in human hepatocytes infected with HAV.

## 2. Materials and Methods

### 2.1. Cell Lines and Reagents

The human hepatoma cell line Huh7 [12] and African green monkey kidney cell line GL37 [13] were used in the present study. Huh7 and GL37 were kindly gifted by Prof. Ralf Bartenschlager (Universität Mainz, Mainz, Germany) and Dr. Tomoko Kiyohara (National Institute of Infectious Diseases, Tokyo, Japan), respectively.

The cells were maintained in Roswell Park Memorial Institute (RPMI) 1640 medium (FUJIFILM Wako Pure Chemical Corporation, Osaka, Japan), Dulbecco’s modified Eagle’s medium (DMEM)-low glucose (1000 mg/L) (Sigma-Aldrich, St. Louis, MO, USA) or DMEM-high-glucose (4500 mg/L) (Sigma-Aldrich) supplemented with 5% fetal bovine serum (FBS, Serana, Pessin, Germany) and 1% penicillin/streptomycin (FUJIFILM Wako). Cells were cultured at 37 °C with a 5% CO_2_ atmosphere.

Zinc acetate, zinc chloride, and zinc sulfate or ribavirin were purchased from FUJIFILM Wako or Sigma-Aldrich, respectively. Zinc acetate, zinc chloride, zinc sulfate, and ribavirin were dissolved in dimethyl sulfoxide (DMSO) (Sigma-Aldrich) for the use in cell culture.

### 2.2. HAV Infection

The cells were infected with HAV HA11-1299 genotype IIIA at a multiplicity of infection (MOI) of 0.01 as previously described [8]. Approximately 1 × 10^6^ cells were infected with 1 × 10^4^ copies/mL HAV RNA. MOI was determined by the methods as previously described [13,14].

Briefly, Huh7 cells were seeded 24 h prior to infection at a density of 0.5 × 10^6^ cells/well in 6-well plates (AGC Techno Glass, Haibara-gun, Shizuoka, Japan). The cells were washed twice with phosphate-buffered saline (PBS; FUJIFILM Wako) and infected with HAV HA11-1299 genotype IIIA in serum-free medium [8]. At 4 h after infection, the cells were washed twice with PBS, and incubated with fresh media. At 24 h after infection, the media was exchanged by DMEM supplemented with 5% FBS with or without zinc compounds at different concentrations. At 48 h after infection, total cellular RNAs were extracted to measure HAV RNA [8].

To examine the effect of a combination of zinc acetate and ribavirin, cells were infected with HAV HA11-1299 genotype IIIA or HAV HM175/18f genotype IB at an MOI of 0.01; HAV HM175/18f genotype IB was provided by Prof. Stanley M Lemon (University of North Carolina at Chapel Hill, Chapel Hill, NC, USA) and Dr. Asuka Hirai-Yuki (National Institute of Infectious Diseases, Tokyo, Japan).

### 2.3. RNA Extraction, cDNA Synthesis, and Quantitative PCR (qPCR) for HAV RNA

Total cellular RNAs were extracted via a QIAshredder (Qiagen GmbH, Hilden, Germany) and RNeasy Mini Kit (Qiagen) as previously described [7]. Reverse transcription was performed using PrimeScript RT reagent (Perfect Real Time; TaKaRa Bio, Kusatsu, Shiga, Japan) at 37 °C for 15 min, followed by incubation at 85 °C for 5 s. For the quantification of HAV RNA, the following primers were used: sense primer, 5′-AGGCTACGGGTGAAACCTCTTAG-3′ and antisense primer, 5′-GCCGCTGTTACCCTATCCAA-3′ [14]. For the quantification of β-actin mRNA, the following primers were used: sense primer, 5′-CAGCCATGTACGTTGCTATCCAGG-3′ and antisense primer, 5′-AGGTCCAGACGCAGGATGGCATG-3′ [15]. Quantitative PCR (qPCR) was performed using PowerSYBR Green PCR Master Mix (Applied Biosystems, Thermo Fisher Scientific, Tokyo, Japan) on a 7500 Fast Real-Time PCR System (Applied Biosystems) or a StepOnePlus Real-Time PCR System (Applied Biosystems) [6]. Real-time PCR assays were performed in triplicate. Huh7 and GL37 cells were analyzed via the ddCt method and standard curve method, respectively.

### 2.4. Half-Maximal Inhibitory Concentration (IC_50_)

IC_50_, which is the concentration of each drug that produces 50% of the maximal inhibitory effect against HAV, was calculated by the formula as previously described [15].

### 2.5. Cell Viability Assay

For the evaluation of cell viability, we performed a dimethylthiazol carboxymethoxyphenyl sulfophenyl tetrazolium (MTS) assay (Promega, Madison, WI, USA) in triplicate. The absorbance of each well at 490 nm was measured with an iMark Microplate Reader (Bio-Rad, Hercules, CA, USA) [8].

### 2.6. Quantitative Detection of the GRP78 Concentration in Conditioned Medium

GRP78 was quantified in conditioned medium from Huh7 cells cultured with a human GRP78/Bip sandwich ELISA kit (Proteintech, Rosemont, IL, USA) [5]. The sensitivity and range were 0.02 ng/mL and 0.156 ng/mL–10 ng/mL, respectively. The absorbance of each well at 450 nm was measured [5].

### 2.7. Statistical Analysis

Statistical analyses were performed with a two-tailed unpaired Student’s *t* test. *p*-value < 0.05 was considered to indicate statistical significance.

## 3. Results

### 3.1. Enhancement of HAV RNA Replication in High-Concentration Glucose Media

**Figure 1:**
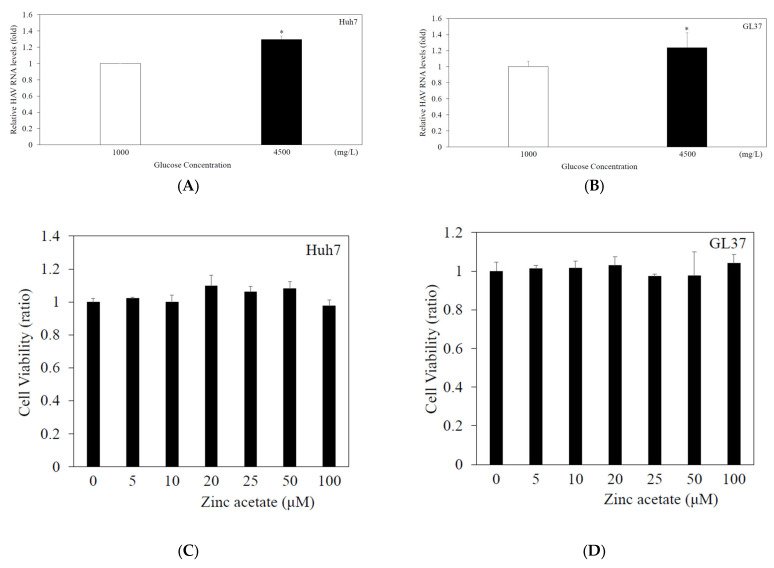
High-concentration glucose enhances HAV HA11-1299 genotype IIIA RNA replication, and zinc acetate has no cytotoxicity. Compared with low-glucose (1000 mg/L) DMEM, high-glucose (4500 mg/L) Dulbecco’s modified Eagle’s medium (DMEM) enhanced HAV HA11-1299 genotype IIIA replication in both Huh7 cells (**A**) and GL37 cells (**B**). HAV RNA was measured by real-time RT-PCR after 48 h of infection. * *p* < 0.05 indicates a statistically significant difference between two groups. Zinc acetate (at the indicated concentration) had no effect on cytotoxicity in either Huh7 cells (**C**) or GL37 cells (**D**). An MTS assay was performed for evaluation after 48 h of incubation. There was no statistically significant difference compared with cells without zinc acetate treatment.

In the present study, Huh7 or GL37 cells were infected with HAV HA11-1299 genotype IIIA at an MOI of 0.01 in high-glucose DMEM (4500 mg/L) or low-glucose DMEM (1000 mg/L). At 48 h of infection, the level of intracellular HAV RNA was measured by real-time RT-PCR. In Huh7 cells, HAV RNA levels were increased 1.30-fold with 4500 mg/L glucose medium compared with those with 1000 mg/L glucose medium (Figure 1A). HAV RNA levels were enhanced by 1.24-fold in GL37 cells cultured with 4500 mg/L glucose compared with those cultured with 1000 mg/L glucose (Figure 1B). Together, these results support a previous report that high-concentration glucose enhances HAV RNA replication in Huh7 cells infected with HAV at 96 h after infection [16].

### 3.2. Zinc Acetate Had No Effect on the Viability of GL37 or Huh7 Cells at 5–100 μM for 48 H

To examine the effects of zinc acetate on cell proliferation (Figure 1C,D), Huh7 and GL37 cells were incubated with different concentrations of zinc acetate (5, 10, 20, 25, 50 and 100 μM) for 48 h, and cell viability was evaluated via the MTS assay. Cellular viabilities were not reduced when Huh7 or GL37 cells were incubated with zinc acetate. We selected concentrations of zinc acetate 10–20 µM for further studies.

### 3.3. Zinc Acetate Significantly Inhibited HAV HA11-1299 Genotype IIIA Replication in Huh7 Cells in High-Concentration Glucose Media

**Figure 2:**
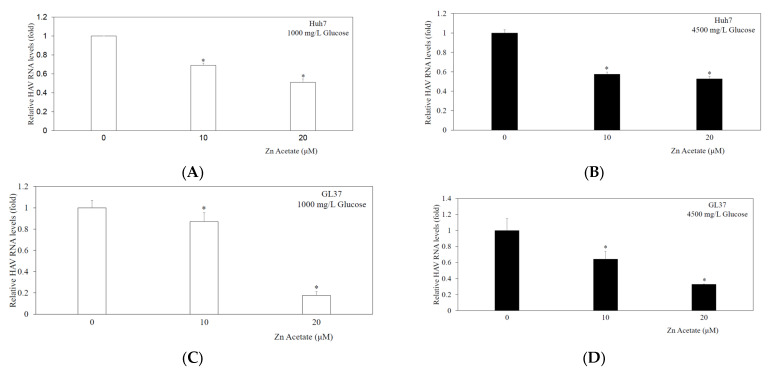
Zinc acetate inhibits HAV HA11-1299 genotype IIIA RNA replication in both Huh7 cells (**A**,**B**) and GL37 cells (**C**,**D**). Cells were cultured in low-glucose Dulbecco’s modified Eagle’s medium (DMEM) (**A**,**C**); or high-glucose DMEM (**B**,**D**). HAV RNA was measured by real-time RT-PCR after 48 h of infection. Zinc acetate was used at the indicated concentrations for 24 h. * *p* < 0.05 indicates a statistically significant difference, compared with those without zinc acetate treatment according to Student’s *t* test.

To examine the effects of zinc acetate on HAV HA11-1299 genotype IIIA replication (Figure 2A,B), Huh7 cells were incubated with different concentrations of zinc acetate (10–20 µM) for 24 h. With 1000 mg/L glucose, HAV replication was reduced to 69% and 51% by 10 μM and 20 μM zinc acetate, respectively, compared with that of the control (100%) (Figure 2A). With 4500 mg/L glucose, HAV replication was reduced to 57% and 53% by 10 μM and 20 μM zinc acetate, respectively, compared with that of the control (100%) (Figure 2B). Thus, in Huh7 cells, despite the high concentration of glucose, zinc sulfate suppressed HAV replication.

### 3.4. Zinc Acetate Significantly Inhibited HAV HA11-1299 Genotype IIIA Replication in GL37 Cells in High-Concentration Glucose Media

To examine the effects of zinc acetate on HAV HA11-1299 genotype IIIA replication (Figure 2C,D), GL37 cells were incubated with different concentrations of zinc acetate (10–20 µM) for 24 h. With 1000 mg/L glucose, HAV replication was reduced to 87% and 18% by 10 μM and 20 μM zinc acetate, respectively, compared with that of the control (100%) (Figure 2C). With 4500 mg/L glucose, HAV replication was reduced to 64% and 33% by 10 μM and 20 μM zinc acetate, respectively, compared with that of the control (100%) (Figure 2D). The IC_50_ value was 13.6 μM. Thus, in GL37 cells, despite the high concentration of glucose, zinc acetate suppressed HAV replication.

### 3.5. Zinc Compounds Significantly Inhibited HAV HA11-1299 Genotype IIIA Replication in Huh7 Cells

**Figure 3:**
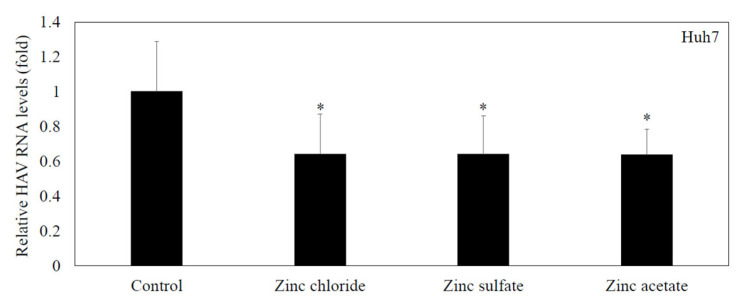
Zinc compounds significantly inhibited HAV HA11-1299 genotype IIIA replication in Huh7 cells. Zinc compounds (zinc chloride, zinc sulfate and zinc acetate) significantly inhibited HAV RNA replication in Huh7 cells compared with cells without treatment. HAV RNA was measured by real-time RT-PCR after 48 h of infection. Zinc compounds were used at 20 μM. * *p* < 0.05 indicates a statistically significant difference, compared with cells without treatment according to Student’s *t* test.

To examine the effects of zinc chloride, zinc sulfate, and zinc acetate on HAV HA11-1299 genotype IIIA replication, Huh7 cells were incubated with zinc chloride, zinc sulfate and zinc acetate at 20 μM for 24 h. HAV replication was reduced to 64%, 64%, and 64% by 20 μM zinc chloride, zinc sulfate and zinc acetate, respectively, compared with the control (100%) (Figure 3). There were no differences in the inhibitory effects of the selected zinc compounds against HAV replication in the present study.

### 3.6. Zinc Acetate Increases GRP78 Concentrations in HAV-Infected Huh7 Cells

We previously reported that zinc sulfate inhibits HAV subgenomic RNA replication in HuhT7 cells, which are Huh7 cells stably expressing T7 polymerase, and that this effect is accompanied by an increase in the GRP78 concentration [5].

**Figure 4:**
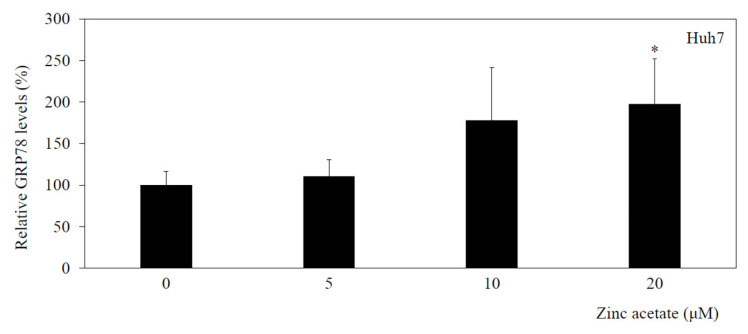
Zinc acetate enhances or inhibits GRP78 concentrations in HAV-infected Huh7 cells. GRP78 was quantified in conditioned medium from cultured Huh7 cells via a human GRP78/Bip sandwich ELISA kit. Black columns, conditioned media from HAV HA11-1299 genotype IIIA-infected cells. * *p* < 0.05 indicates a statistically significant difference, compared with cells without treatment according to Student’s *t* test.

In the present study, the concentration of GRP78 in conditioned medium from zinc acetate-treated Huh7 cells infected with HAV was determined by ELISA (Figure 4). In Huh7 cells infected with HAV, the GRP78 concentration in the conditioned medium gradually increased with increasing zinc acetate concentration.

### 3.7. Effects of Zinc Acetate With or Without Ribavirin on HAV Replication in Huh7 Cells

**Figure 5:**
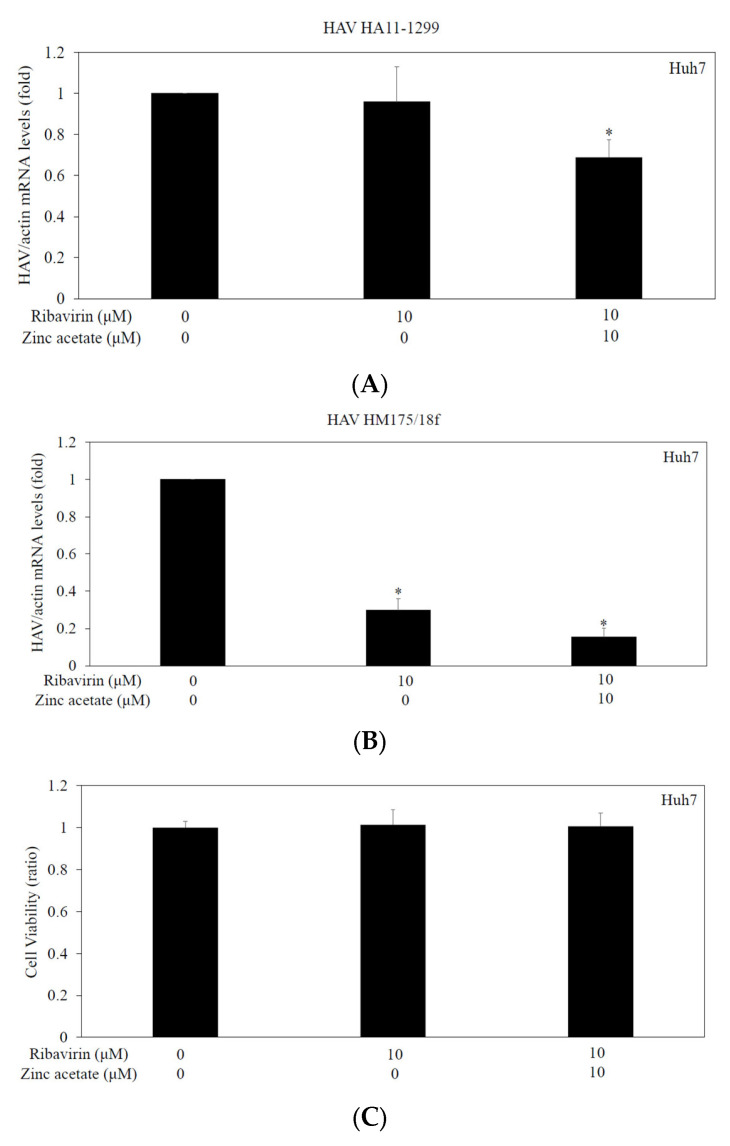
Effects of zinc acetate with or without ribavirin on HAV replication in Huh7 cells. Cells infected with HAV HA11-1299 genotype IIIA (**A**) or HAV HM175/18f genotype IB (**B**) were used. HAV RNA was determined by real-time RT-PCR after 48 h of infection. Zinc acetate and ribavirin were used at the indicated concentrations for 24 h. * *p* < 0.05 indicates a statistically significant difference, compared with those without treatment according to Student’s *t* test. (**C**) The combination of ribavirin and zinc acetate (at the indicated concentrations) had no effect on cell viability of Huh7 cells. An MTS assay was performed for evaluation after 72 h of incubation. There were no statistically significant differences, compared with cells without treatment according to Student’s *t* test.

To further determine the effects of the combination of zinc acetate with ribavirin on HAV HA11-1299 genotype IIIA or HAV HM175/18f genotype IB replication in Huh7 cells, we performed an HAV infectivity assay (Figure 5). Huh7 cell monolayers in 6-well culture plates were infected with HAV at 0.01 MOI and treated with 0 or 10 μM ribavirin and 0 or 10 μM zinc acetate for 24 h.

Suppression of both the HAV HA11-1299 genotype IIIA and HAV HM175/18f genotype IB replication by the combination of ribavirin and zinc acetate was greater than that of ribavirin alone or the untreated control (Figure 5A,B). No effects of the combination of ribavirin and zinc acetate on the cell viability of Huh7 cells were observed (Figure 5C).

## 4. Discussion

GRP78 is an antiviral protein that prevents HAV replication [16,17]. We previously verified the enhancement of HAV replication under GRP78 functional inhibition experiments (siRNA knockdown or knockout by clustered regularly interspaced short palindromic repeats (CRISPR)/CRISPR-associated protein 9 (Cas9)-mediated genome editing) [17], and the inhibition of HAV replication with GRP78 overexpression by [16]. In the present study, we observed that zinc acetate inhibited HAV replication, along with the enhancement of GRP78 protein expression.

We have previously shown that zinc sulfate, zinc chloride and Japanese rice-koji miso have inhibitory effects on HAV replication [5,6,7,8,18] and that zinc sulfate increases GRP78 expression in HAV-infected hepatocytes [5]. Zinc chloride decreases the expression of mitogen-activated protein kinase 3 (MAP2K3), resulting in inhibiting HAV replication [7]. We observed that zinc acetate inhibited HAV HA11-1299 replication in both Huh7 and GL37 cells, although zinc acetate is useful for the treatment of Wilson’s disease and zinc deficiency in clinical settings [19,20]. However, in the present study, we did not observe any differences in the inhibitory effects of zinc sulfate, zinc chloride, or zinc acetate on HAV HA11-1299 replication.

In human, a serum zinc level < 60 μg/dL, 60–80 μg/dL and >80 μg/dL indicate zinc deficiency, marginal zinc deficiency, and the normal zinc status, respectively [20]. It is expected that 10–20 μM zinc acetate includes approximately 60–120 μg/dL zinc, because the molecular weight of zinc acetate and zinc are 183.5 g/mol and 65.38 g/mol, respectively. These are reasons for selecting 10–20 µM zinc acetate concentrations in the present study.

We previously reported that high concentrations of glucose enhanced HAV replication, in association with a reduction in GRP78 expression [16]. Nakao et al. [21] reported that diabetes mellitus was more common among deceased patients with HAV-ALF than among rescued patients with HAV-ALF (29% vs. 8%; *p* < 0.05). Kumar et al. [22] reported that diabetes leads to adverse outcomes in patients with metabolic dysfunction associated with fatty liver disease-related ACLF. We reconfirmed that a high concentration of glucose enhances HAV replication in both cultured Huh7 and in GL37 cells.

The increase in HAV replication under high-glucose conditions is modest (1.30-fold in Huh7, 1.24-fold in GL37) (Figure 1A,B). Glucose metabolism plays an important role in driving immune responses against viral infections [23]. HAV may evolve mechanisms to use host metabolism, by hijacking glucose-dependent pathways, and HAV may continue replication and modify immune responses.

Furthermore, in the present study, zinc acetate inhibited HAV replication in both low- and high-glucose media. These results indicate that zinc acetate may be useful for the treatment of severe acute hepatitis, although it may be ineffective for the treatment of end-stage liver diseases [19].

We also observed that zinc acetate increased the GRP78 concentration in the context of HAV infection. While GRP78 upregulation is highlighted, the exact pathway by which zinc acetate induces GRP78 and inhibits HAV remains unclear. It is possible that zinc deficiency could lead to oxidative stress, endoplasmic stress, apoptosis in hepatocytes and inflammation in the liver [24]. Endoplasmic stress is associated with HAV replication [8,17,25].

GRP78 can increase the activation of the interferon/JAK-STAT pathway during influenza A virus infection [11]. Zinc acetate may be more useful for treating diseases in which GRP78 is inhibited [18]. Interferon signaling plays an important role in controlling HAV replication in vivo [26]. Further studies may be needed to show whether GRP78 is the main factor or just one part of a wider antiviral effect of zinc acetate.

We also observed that the combination of zinc acetate with ribavirin affected HAV HA11-1299 genotype IIIA or HAV HM175/18f genotype IB replication in Huh7 cells. Although ribavirin is useful for the inhibition of HAV replication [27,28], it is associated with adverse events, such as hemolytic anemia. The addition of zinc acetate to ribavirin therapies may decrease the dose of ribavirin that patients receive, reducing the occurrence of adverse events.

GRP78 is an antiviral factor in several viral infections, such as influenza A virus [11], hepatitis B virus [29], hepatitis C virus [30] and Japanese encephalitis virus (JEV) [31]. Several reports have shown that GRP78 enhances interferon signaling during viral infection, including HAV infection [7,11,29,30]. Silencing GRP78 expression in persistently JEV-infected cells led to C/EBP homologous binding protein (CHOP) induction, followed by a severe reduction in cell viability [31]. In general, HAV infection does not induce cellular apoptosis [32], except for some cases [33]. However, further studies on the mechanism underlying the association between GRP78 and anti-HAV agents are needed.

In general, patients with ACLF often have advanced chronic liver diseases, such as liver cirrhosis [22]. Patients with cirrhosis possess hypozincemia [2,20]. It may be useful for ACLF-patients infected with HAV to be treated by zinc compounds.

Zinc chloride, zinc sulfate, and zinc acetate which are water soluble, include 48%, 28%, 30% of element zinc content, respectively [34]. Zinc is primarily absorbed at duodenum and jejunum [35]. It was reported that the highest zinc plasma concentrations were observed with zinc acetate at a low pH in the stomach [36]. Japan’s Health Insurance system has approved zinc acetate hydrate for the safe treatment of patients with zinc deficiency. We showed that zinc acetate inhibits HAV replication, and our studies shed new light on the lack of effective treatments for hepatitis A, although there have been several reports about anti-HAV compounds [37,38,39,40,41,42,43,44,45,46].

In the present study, high-glucose medium was used to mimic HAV severity in diabetic patients, but we do not know whether this culture condition accurately reflects the intracellular hepatic environment in actual diabetic patients. Particularly, diabetic patients have complex metabolic abnormalities including not only simple hyperglycemia but also insulin resistance, increased inflammatory cytokines, and oxidative stress. This may be one of the limitations of study.

## 5. Conclusions

We demonstrated that zinc acetate inhibits HAV replication, which is accompanied by increased GRP78 expression. The inhibitory effect of zinc acetate on HAV replication may be related to a known mechanism [47,48] without any serious toxicity to hepatocytes or renal cells. However, further understanding of the complex mechanism by which zinc acetate affects the expression of GRP78 in the context of HAV infection is needed. Bioavailability of zinc acetate could be a key practical challenge in patients with cirrhosis. Further studies at this point are needed in animal models.

## Data Availability

The raw data supporting the conclusions of this article will be made available by the authors on request.

## Abbreviations

The following abbreviations are used in this manuscript:

HAV	hepatitis A virus
ACLF	acute-on-chronic liver failure
GRP78	glucose-regulated protein 78
ALF	acute liver failure
IRES	internal ribosomal entry site
NTR	nontranslational region
ER	endoplasmic reticulum
BiP	binding immunoglobulin protein
HSPA5	heat shock protein family A member 5
RNA-Seq	transcriptome sequencing
JAK	Janus kinase
STAT	signal transducer and activator of transcription
DMEM	Dulbecco’s modified Eagle’s medium
IC_50_	half-maximal inhibitory concentration
MTS	dimethylthiazol carboxymethoxyphenyl sulfophenyl tetrazolium
MOI	multiplicity of infection
MAP2K3	mitogen-activated protein kinase 3
poly(I:C)	polyinosinic–polycytidylic acid
